# Atypical Cartilaginous Tumors: Trends in Management

**DOI:** 10.5435/JAAOSGlobal-D-21-00277

**Published:** 2021-12-16

**Authors:** Matthew E. Wells, Benjamin R. Childs, Michael D. Eckhoff, Rajiv Rajani, Benjamin K. Potter, Elizabeth M. Polfer

**Affiliations:** From the William Beaumont Army Medical Center, El Paso, TX (Dr. Wells, Dr. Childs, Dr. Eckhoff, and Dr. Polfer); the Texas Tech University Health Sciences Center El Paso, El Paso, TX (Dr. Wells, Dr. Childs, Dr. Eckhoff, and Dr. Rajani); and the Uniformed Services University of the Health Sciences, Bethesda, MD (Dr. Potter and Dr. Polfer).

## Abstract

**Introduction:**

Chondrosarcomas are the most common primary bone malignancy in adults within the United States. Low-grade chondrosarcomas of the long bones, now referred to as atypical cartilaginous tumors (ACTs), have undergone considerable changes in recommended management over the past 20 years, although controversy remains. Diagnostic needle biopsy is recommended only in ambiguous lesions that cannot be clinically diagnosed with a multidisciplinary team. Local excision is preferred due to better functional and equivalent oncologic outcomes. We sought to determine whether these changes are reflected in reported management of ACTs.

**Methods:**

The National Cancer Database (NCDB) 2004 to 2016 was queried for ACTs of the long bones. Reported patient demographics and tumor clinicopathologic findings were extracted and compared between patients who underwent local excision versus wide resection.

**Results:**

We identified 1174 ACT patients in the NCDB. Of these, 586 underwent local excision and 588 underwent wide resection. No significant differences were found in patient demographics. No significant change was found in the reported percentage of diagnostic biopsies or wide resections performed over time. After multivariate regression, the single greatest predictor of performing wide resection on an ACTs was presenting tumor size.

**Discussion:**

Evaluation of the NCDB demonstrated that despite changes in the recommended management of ACTs, there has not been a significant change in surgical treatment over time. Surgeons have been performing diagnostic biopsies and wide resections at similar to historical rates. Persistency of these practices may be due to presenting tumor size, complex anatomic location, uncertainty of underlying tumor grade, or patient choice as part of clinical shared decision making. The authors anticipate that the rate of biopsies and wide resections performed will decrease over time as a result of improvements in advanced imaging and the implementation of recently updated clinical practice guidelines.

Chondrosarcomas are the most commonly encountered primary bone tumors of adulthood within the United States.^1^ These tumors most often occur during the fourth and fifth decades of life in the long bones such as the humerus, femur, or tibia.^2,3,4,5^ The incidence of low-grade chondrosarcomas has increased over time secondary to both an aging population and incidental findings from increased diagnostic imaging.^5^ In the World Health Organization's newest edition of soft-tissue and bone tumors, the terminology of atypical cartilaginous tumor (ACT) is reserved for low-grade (grade 1) chondrosarcomas located within the appendicular skeleton both in long and short tubular bones.^6^ The terminology of grade 1 chondrosarcoma is reserved for tumors of the axial skeleton (pelvis, scapula, and skull base flat bones) to reflect the poorer clinical outcome of these tumors at these sites.^5-7^

ACTs are often seen as ambiguous lesions with cartilaginous appearance on standard imaging similar to that of an enchondroma. However, ACTs often display more aggressive and destructive features than their definitive benign counterparts. An enchondroma is a well-defined lytic lesion with stippled calcifications in the pattern of rings and arcs within the metaphysis or metadiaphyseal region of long bones.^8^ Some enchondromas can have an aggressive radiographic appearance near identical to ACTs with larger intramedullary involvement and moderate endosteal scalloping.^9^ Thus, differentiating between a benign enchondroma and an ACT with malignant potential can be notably difficult based on radiographic imaging alone. Historically, this conundrum required a tissue biopsy to make a distinction. However, utilization of dynamic gadolinium chelate-enhanced MRI and/or positron emission tomography CT (PET-CT) can aid differentiating between enchondroma and an ACT,^10,11,12,13^ negating the need for biopsy. If radiographic or advanced imaging demonstrates gross cortical erosion or soft-tissue infiltration, the lesion is approached as an intermediate- or high-grade chondrosarcoma until proven otherwise.^9^ Therefore, differentiating between an ACT and a high-grade chondrosarcoma is often less controversial. However, if there is still ambiguity, differentiation between these low-grade and high-grade lesions may also be conferred with advanced imaging,^10,11,12,13^ again negating the need for biopsy.

Therefore, because of high concordance of biopsy sample and presumed diagnosis on imaging studies alone, a diagnostic biopsy is often not necessary.^14-16^ The utilization of advanced imaging and collaboration between multidisciplinary teams allows for high diagnostic accuracy.^9,11,15-17^ In addition, although treatment of an ACT with wide resection was often favored in the past due to concern for malignant potential,^3^ contemporary literature recommends extended intralesional excision with curettage, adjunctive high-speed burring, and bone void augmentation.^18,19,20,21,22,23,24,25^ The more aggressive intermediate- and high-grade chondrosarcomas, especially within the pelvis, have high malignant potential and are treated with wide resection and reconstruction.^24,25^

With recommendations shifting away from biopsy and wide resection toward advanced imaging and less aggressive surgical management, this article investigates the trends in ACT management from 2004 to 2016 using the National Cancer Database (NCDB). In addition, we sought to identify factors that were associated with diagnostic biopsy and type of definitive surgery performed.

## Methods

The NCDB includes 34 million patient records with a 90% follow-up during a 5-year period.^26^ Although participating cancer centers represent approximately 30% of hospitals within the United States, the NCDB encompasses approximately 70% of all patients newly diagnosed with cancer.^27,28^ Our institutional review board determined that this was nonhuman subject research and did not require formal review. ACT cases were extracted from the bone and joint participant use file from the NCDB (2004 to 2016) using International Classification of Diseases for Oncology (ICD-O) code of 9220 (cartilage-based tumors), behavior code 3 (chondrosarcoma), grade code 1, and long bone location. A biopsy performed was defined as either incisional, needle, or aspiration methodology.

Demographic and outcome variables including age, sex, race, insurance status, and treatment facility type were reported. Patient and tumor-specific measures included Charlson-Deyo score,^29^ history of previous cancers, American Joint Committee on Cancer tumor size (T), reported diagnostic biopsy procedure, and time from diagnosis to definitive surgery. These variables were categorized as local excision versus wide resection. The NCDB prohibits the reporting of parameter counts fewer than 10. Lymph node extension and metastatic staging were unable to be reported due to these parameter restrictions. A Kendall tau-b correlation was performed to determine trends in the percent of patients with a long bone ACT who underwent an initial diagnostic biopsy before definitive surgical management and the percentage of patients who underwent wide resection over time. Independent samples *t*-tests were used to compare means of continuous variables, and *P* values less than 0.05 were considered to represent a significant difference. For variables with Levene test for equality of variances greater than 0.05, equal variance was assumed. The Pearson χ^2^ test *P* values less than 0.008 were considered to represent a significant difference in categorical variables after Bonferroni correction. Binary logistic regressions were used to calculate odds ratios. Multivariate binary logistic regression was attempted for all significant variables and reported. All analysis was performed in SPSS version 25 (IBM Released 2018. IBM SPSS Statistics for Macintosh, Version 25.0: IBM).

## Results

We identified 1174 ACT patients in the NCDB. Of these, 586 underwent local excision, and 588 underwent wide resection. No significant difference was found between age, sex, race, insurance status, facility treatment type, Charleson-Deyo score, and history of previous cancers between the two groups (Supplemental Table 1, http://links.lww.com/JG9/A174). A significant association was found between the American Joint Committee on Cancer tumor size and the decision to perform a diagnostic biopsy (T1 31.8% versus T2 43.9%, *P* = 0.001). Patients who underwent diagnostic biopsy before definitive surgery were more likely to be treated with local excision than wide resection (52.7% versus 42.7%, respectively, *P* = 0.003; Supplemental Table 2, http://links.lww.com/JG9/A175). Tumors staged as T1 preferentially underwent local excision procedures compared with tumors staged T2 (53% versus 33.8%, respectively, *P* < 0.001; Supplemental Table 2, http://links.lww.com/JG9/A175). Subgroup analysis comparing the percentage of patients with T1 or T2 tumors undergoing diagnostic biopsies or wide resection from 2004 to 2016 also remained unchanged over time (T1 *P* = 0.393 and *P* = 0.805; T2 *P* = 0.105 and *P* = 0.405, respectively). No significant difference was found in the time from diagnosis to definitive surgery.

After multivariable regression, only ACT tumor size was statistically significant for predicting wide resection after correction for reported biopsy procedures (odds ratio, 2.1; 95% CI, 1.5 to 3.0, *P* <0.001; Supplemental Table 2, http://links.lww.com/JG9/A175). There was no significant change year to year in percent of patients who had a diagnostic biopsy (*P* = 0.794, Supplemental Figure 1, http://links.lww.com/JG9/A176) or wide resection (*P* = 0.53; Supplemental Figure 2, http://links.lww.com/JG9/A177).

## Discussion

The recommended management of long bone ACTs has assumed less aggressive surgical management over the last 20 years. Orthopaedic surgeons often chose biopsy and/or wide resection over extended intralesional excision of radiographically determined ACTs.^30^ Presumed diagnosis of ACT based on advanced imaging and clinical examination without a diagnostic biopsy is considered safe and appropriate.^11,14,15^ Primary surgical management changes have shifted toward less aggressive treatment to include extended intralesional curettage, adjunctive high-speed burring, and bone void augmentation.^11,18,22,24,25^ However, this analysis of the NCDB shows that these changes are not necessarily reflected in the percentage of patients undergoing diagnostic biopsy or wide resections performed per year. The lack of change may be due to the lag of treatment implementation after only recently updated clinical practice guidelines, presenting tumor size, anatomic location, uncertainty of underlying tumor grade, or patient choice as part of clinical shared decision making.

The percentage of patients who underwent a diagnostic biopsy did not change over time. It should be noted that not all cartilaginous tumors show classic radiographic features and experienced musculoskeletal oncologists have often relied on cortical involvement on radiographs, neoplasm size, and presence of pain to establish a preoperative diagnosis.^30^ Advanced imaging studies continue to improve as dynamic gadolinium chelate-enhanced MRI and/or PET-CT to aid differentiating between cartilaginous tumors.^10,11,12,13^ However, if ambiguity remains and a cartilaginous tumor cannot reliably differentiated based on radiographic and advanced imaging with collaboration of a multidisciplinary team, a biopsy is still warranted.

Among patients who underwent a biopsy with a resultant diagnosis of ACT, smaller (T1) tumors were marginally more likely to be treated with extended intralesional excision, reflecting some appreciable acceptance in less invasive treatment options. However, larger (T2) tumors continued to undergo wide resection considerably more often than extended intralesional excision. It can be assumed that this was likely due to concern of diagnostic error or variability in the performed biopsy as large chondrosarcomas may be heterogeneous and can have variable low- and high-grade components. However, recent literature supports a high diagnostic concordance rate among biopsy and ultimately excised tumor for long bone chondrosarcomas.^22^ Therefore, in the setting of a well-established ACT, it is safe and effective to perform extended intralesional curettage without a biopsy and certainly after biopsy. Multidisciplinary consultation cannot be underemphasized.

The surgical management of ACTs has not significantly changed over time. This may have resulted in patients being treated with more aggressive surgical modalities than necessary. Multiple previous systematic reviews, meta-analyses, and comparative studies have been performed comparing intralesional excision and wide resection for ACT.^2,18,19,20,21,22,23^ There is a lower rate of complications in patients treated with intralesional excision treated patients than the wide resection, consistent with other studies.^23,31^ Specific comparative studies have also found improved Musculoskeletal Tumor Society score and better self-rated function with intralesional excision compared with wide resection.^18,20^ Collectively, these findings support the treatment of ACT with intralesional excision over wide resection. Regardless, it is crucial to consider the entire clinical picture as the location, size, and diagnosis have implications regarding prognosis and management.^24,25^ Again, the importance of establishing a multidisciplinary team cannot be overstated as together these teams determine the most appropriate courses of action for the management of musculoskeletal malignancies, particularly in the setting of ACTs. Although some physicians remain concerned about the development of metastatic spread from an ACT, the probability of developing metastases due to ACT is relatively low and reported as 5% at 5 years and 8% at 10 years.^31^ Chen et al^23^ recently performed a meta-analysis and found no difference in ACT local recurrence or metastasis between intralesional excision and wide resection even when accounting for specific anatomic locations (humerus, tibia, and femur). Therefore, patients with larger (T2) ACTs may still be receiving more aggressive surgical treatment than necessary.

There are a number of limitations with the use of large databases including the NCDB. A larger proportion of individuals younger than 65 years are captured in the database.^26^ Analysis is limited to variables provided by the database and may not include demographic or treatment considerations such as patient employment status, hospital size, types of imaging used for staging purposes, or the reported cause of death. Specific to this study, we were not able to determine whether there was utilization of dynamic gadolinium chelate-enhanced MRI and/or PET-CT^10,11,12,13^ to obtain and underlying diagnosis. Ultimately, the NCDB is a database, and although there are highly standardized reporting protocols with a number of integrity checks and quality assurance measures to ensure accuracy of reporting, human error and ambiguous diagnoses must be considered in interpretation of results. In addition, there has been growing evidence over the past 20 years for the use of advanced imaging and local excision over the more traditional biopsy and wide resection treatment. However, the World Health Organization classification for ACTs and updated clinical practice guidelines only recently changed, and long-term changes in the NCDB may not be appreciated at this time.

Notable controversy has surrounded the diagnosis and surgical management of long bone ACTs in years past. Contemporary recommendations for the surgical management of ACTs of the extremities do not necessitate biopsy and include extended intralesional excision with curettage, adjunctive high-speed burring, and bone void augmentation. The NCDB was queried and showed that surgeons have been choosing to obtain a biopsy and/or perform wide resection over extended intralesional curettage in patients diagnosed with ACT of the extremities. This may have resulted in potential for more aggressive treatment than was necessary in some cases. Wide resection procedures were associated with larger initial presenting tumor sizes, however may also be attributed to ambiguity of biopsy samples, periarticular tumor locations necessitating reconstruction, or other patient-specific considerations. Ultimately, each patient should be considered on a case-to-case basis. The authors anticipate that the rate of biopsies and wide resections performed for ACTs will decrease over time as a result of improvements in advanced imaging and the implementation of recently updated clinical practice guidelines. It behooves orthopaedic surgeons to establish a multidisciplinary team early on in the diagnosis and evaluation steps, as this may lead to less aggressive surgical treatments with overall better functional outcomes.

## Supplementary Material

SUPPLEMENTARY MATERIAL
