# Supplementary material for: Atypical Cartilaginous Tumors: Trends in Management
Source: J Am Acad Orthop Surg Glob Res Rev. 2021 Dec 16;5(12):e21.00277. doi: 10.5435/JAAOSGlobal-D-21-00277 (PMC8683228; doi:10.5435/JAAOSGlobal-D-21-00277)
Supplement: SUPPLEMENTARY MATERIAL [file jagrr-5-e21.00277-s001.docx]

| **Supplemental Table 1.** Demographic and facility characteristics of reported patients who underwent surgical removal of a long bone ACT. |
| --- |
| \|  \|  \| **Extended Intralesional Excision** \| **Wide**  **Resection** \| **p** \| \| --- \| --- \| --- \| --- \| --- \| \|  \|  \| **(n=586)** \| **(n=588)** \|  \| \| **Age** \| Years \| 49.3 ± 14.3 \| 49.7 ± 15.8 \| 0.716 \| \| **Gender** \| Female \| 371 \| 341 \| 0.062 \| \| (52.1%) \| (47.9%) \| \| Male \| 215 \| 247 \| \| (46.5%) \| (53.5%) \| \| **Race** \| White \| 512 \| 512 \| 0.427 \| \| (50.0%) \| (50.0%) \| \| Other \| 74 \| 76 \| \| (49.3%) \| (50.7%) \| \| **Insurance Status** \| Not insured \| 26 \| 27 \| 0.888 \| \| (49.1%) \| (50.9%) \| \| Insured \| 548 \| 547 \| \| (50.0%) \| (50.0%) \| \| **Facility type** \| Community Hospital \| 59  (49.2%) \| 61  (50.8%) \| 0.69 \| \| Academic Center \| 386  (51.1%) \| 369  (48.9%) \| \| **Charlson Deyo Score** \| 0 \| 480 (49.3%) \| 493 (50.7%) \| 0.239 \| \| 1 \| 89 (55.3%) \| 72 (44.7%) \| \| ≥2 \| 17 (42.5%) \| 23  (57.5%) \| \| **Hx of prior cancers** \| No \| 523 (50.0%) \| 522 (50.0%) \| 0.795 \| \| Yes \| 63 (48.8%) \| 66 (51.2%) \| |
|  |
