# Supplementary material for: Atypical Cartilaginous Tumors: Trends in Management
Source: J Am Acad Orthop Surg Glob Res Rev. 2021 Dec 16;5(12):e21.00277. doi: 10.5435/JAAOSGlobal-D-21-00277 (PMC8683228; doi:10.5435/JAAOSGlobal-D-21-00277)
Supplement: SUPPLEMENTARY MATERIAL [file jagrr-5-e21.00277-s002.docx]

| **Supplemental Table 2.** Clinical characteristics and staging procedures of reported long bone ACTs that underwent surgical removal. |
| --- |
| \|  \| \|  \| \| **Extended Intralesional Excision** \| \| **Wide**  **Resection** \| \| **p** \| **Odd's Ratio*** \| \| \| --- \| --- \| --- \| --- \| --- \| --- \| --- \| --- \| --- \| --- \| --- \| \| \|  \|  \| \| **(n=586)** \| \| **(n=588)** \| \|  \| \| \|  \| \| \| **AJCC Primary Tumor Size (T)** \| \| T1 \| \| 296 (53.0%) \| \| 263 (47.0%) \| \| <0.001 \| 2.1 [1.5-3.0] \| \| \| T2 \| \| 67 (33.8%) \| \| 131 (66.2%) \| \| \| **Diagnostic Procedure** \| \| Biopsy Performed \| \| 448 (52.7%) \| \| 402 (47.3%) \| \| 0.003 \|  \| \| \| No biopsy Performed \| \| 132 (42.7%) \| \| 177 (57.3%) \| \| \| **Time from Dx to Definitive Surgery** \| \| Days \| \| 17.0 ± 52.8 \| \| 21.2 ± 35.7 \| \| 0.115 \|  \| \| |
| * After multivariable regression, only tumor size was considered significant when factored for reported biopsy procedures performed. |
