# Supplementary material for: Atypical Cartilaginous Tumors: Trends in Management
Source: J Am Acad Orthop Surg Glob Res Rev. 2021 Dec 16;5(12):e21.00277. doi: 10.5435/JAAOSGlobal-D-21-00277 (PMC8683228; doi:10.5435/JAAOSGlobal-D-21-00277)
Supplement: SUPPLEMENTARY MATERIAL [file jagrr-5-e21.00277-s003.pdf]

Supplemental Figure 1. There was no change in the percent of patients who had an initial diagnostic biopsy performed by year.

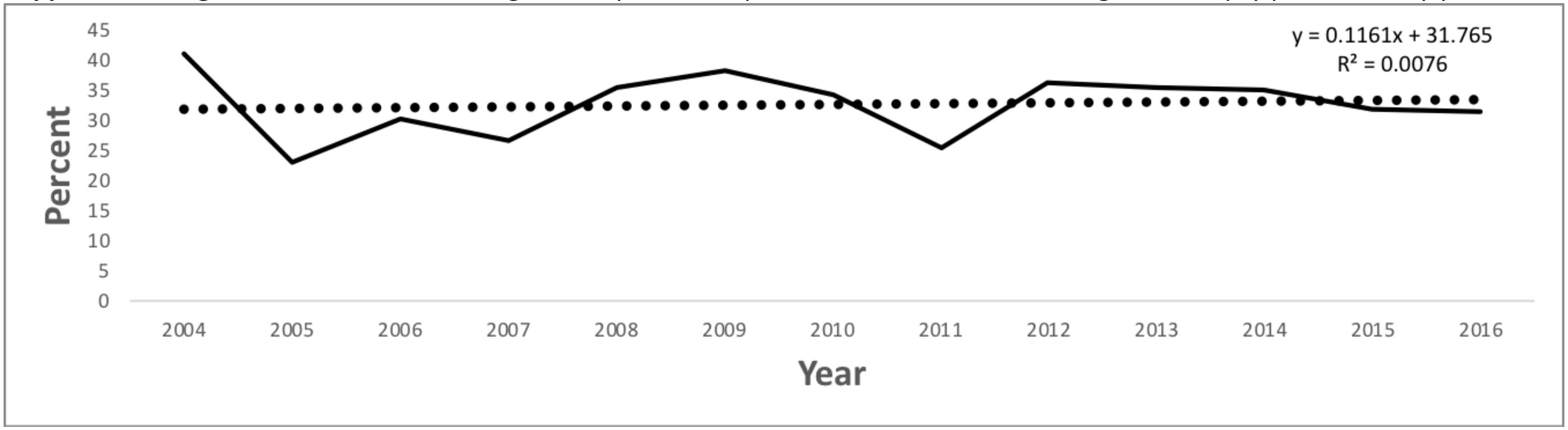

A Kendall's tau-b correlation was performed to determine trends in the percent of patients with a long bone atypical cartilaginous tumor who underwent an initial diagnostic biopsy prior to definitive surgical management between 2004 and 2016. There was no statistically significant change in percent of patients who had a diagnostic biopsy ($p = 0.794$).
