# Supplementary material for: Atypical Cartilaginous Tumors: Trends in Management
Source: J Am Acad Orthop Surg Glob Res Rev. 2021 Dec 16;5(12):e21.00277. doi: 10.5435/JAAOSGlobal-D-21-00277 (PMC8683228; doi:10.5435/JAAOSGlobal-D-21-00277)
Supplement: SUPPLEMENTARY MATERIAL [file jagrr-5-e21.00277-s004.pdf]

Supplemental Figure 2. There was no change in the percent of patients who undergo wide resection by year.

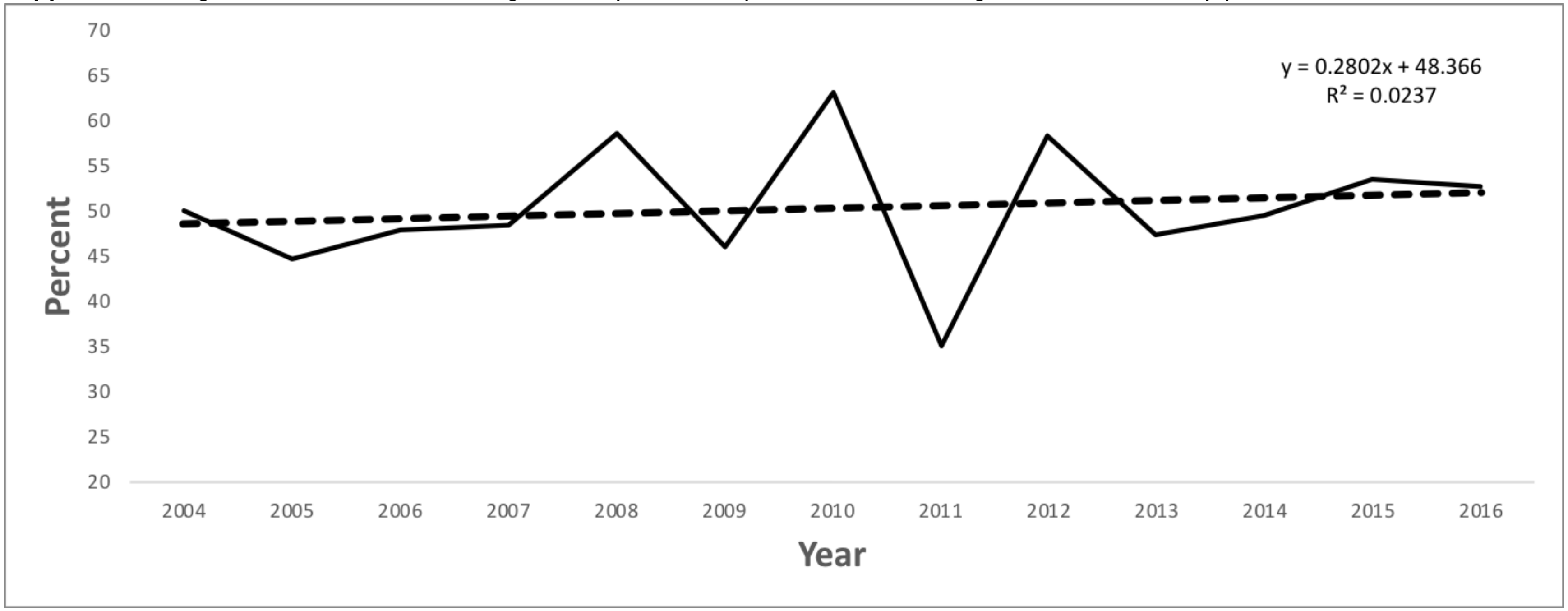

A Kendall's tau-b correlation was performed to determine trends in the percent of patients with a long bone atypical cartilaginous tumor who underwent wide resection procedures performed between 2004 and 2016. There was no statistically significant change in percent of patients who underwent wide resection ($p = 0.530$).
